# Efficacy of Violet-Blue (405 nm) LED Lamps for Disinfection of High-Environmental-Contact Surfaces in Healthcare Facilities: Leading to the Inactivation of Microorganisms and Reduction of MRSA Contamination

**DOI:** 10.3390/pathogens12111338

**Published:** 2023-11-10

**Authors:** Davide Amodeo, Pietro Manzi, Isa De Palma, Alessandro Puccio, Nicola Nante, Mariella Barcaccia, Daniele Marini, Donatella Pietrella

**Affiliations:** 1 Department of Medical Biotechnology, University of Siena, 53100 Siena, Italy; isa.depalma@student.unisi.it; 2 Hospital of Santa Maria di Terni, 05100 Terni, Italy; dottormanzi@gmail.com; 3 Department of Molecular and Developmental Medicine, University of Siena, 53100 Siena, Italy; alessandropuccio@hotmail.com (A.P.); nicola.nante@unisi.it (N.N.); 4 Microbiology Unit, Perugia General Hospital, 06100 Perugia, Italy; mariella.barcaccia@ospedale.perugia.it; 5 Medical Microbiology Section, Department of Medicine and Surgery, University of Perugia, 06100 Perugia, Italy; daniele.marini2@studenti.unipg.it (D.M.); donatella.pietrella@unipg.it (D.P.)

**Keywords:** violet-blue light, disinfection, UV radiation, HAI, MRSA, surface contamination, LED technology

## Abstract

Effective disinfection procedures in healthcare facilities are essential to prevent transmission. Chemical disinfectants, hydrogen peroxide vapour (HPV) systems and ultraviolet (UV) light are commonly used methods. An emerging method, violet-blue light at 405 nm, has shown promise for surface disinfection. Its antimicrobial properties are based on producing reactive oxygen species (ROS) that lead to the inactivation of pathogens. Studies have shown significant efficacy in reducing bacterial levels on surfaces and in the air, reducing nosocomial infections. The aim of this study was to evaluate the antimicrobial effectiveness of violet-blue (405 nm) LED lamps on high-contact surfaces in a hospital infection-control laboratory. High-contact surfaces were sampled before and after 7 days of exposure to violet-blue light. In addition, the effect of violet-blue light on MRSA-contaminated surfaces was investigated. Exposure to violet-blue light significantly reduced the number of bacteria, yeasts and moulds on the sampled surfaces. The incubator handle showed a low microbial load and no growth after irradiation. The worktable and sink showed an inconsistent reduction due to shaded areas. In the second experiment, violet-blue light significantly reduced the microbial load of MRSA on surfaces, with a greater reduction on steel surfaces than on plastic surfaces. Violet-blue light at 405 nm has proven to be an effective tool for pathogen inactivation in healthcare settings Violet-blue light shows promise as an additional and integrated tool to reduce microbial contamination in hospital environments but must be used in combination with standard cleaning practices and infection control protocols. Further research is needed to optimise the violet-blue, 405 nm disinfection method.

## 1. Introduction

Healthcare-associated infections (HAIs) continue to be a major problem in healthcare facilities, and the role of healthcare workers in transmitting these infections is well documented [1,2]. Healthcare workers, including doctors, nurses and other staff members, can act as vectors for transmitting pathogens within healthcare facilities. The nature of their work, which involves close and frequent contact with patients, exposes them to an increased risk of acquiring and spreading infections [3,4].

A significant route of transmission is through the contaminated hands of healthcare workers. Proper hand hygiene practices are essential to prevent the spread of infections, but adherence to hand hygiene protocols remains suboptimal in many healthcare facilities [5,6,7]. Studies have consistently shown an association between poor hand hygiene and increased HAI rates. A systematic review by Erasmus et al. [8] showed that increased adherence to hand hygiene significantly reduces the incidence of HAIs.

In addition, healthcare personnel may contribute to transmitting infections through inappropriate use and handling of medical devices. For example, failure to observe proper aseptic techniques during the insertion and maintenance of invasive devices, such as urinary catheters or central lines, can introduce pathogens into the patient’s body [9]. A study by Mody et al. [10] highlighted the need for comprehensive educational programmes aimed at healthcare workers to improve their knowledge and adherence to infection prevention practices.

In addition, healthcare workers themselves can become infected and act as a source of transmission if they are unaware of their infectious status. Some infectious diseases, such as tuberculosis or respiratory infections, pose a risk to healthcare workers and patients. Implementing screening programmes, vaccination and regular health checks for healthcare workers can help to detect and treat infections early and reduce the risk of transmission. The Centres for Disease Control and Prevention (CDC) and the World Health Organization (WHO) provide guidelines and recommendations for the health and safety of healthcare workers [11,12].

The prevention of HAIs requires the implementation of effective disinfection procedures in healthcare facilities [13]. Several methods can be used to reduce the risk of transmission, including the use of chemical disinfectants, hydrogen peroxide vapour (HPV) systems and ultraviolet (UV) radiation. Chemical disinfectants, such as quaternary ammonium compounds, bleach and alcohol-based solutions, are commonly used for surface disinfection [14]. A study by Weber et al. [15] evaluated the efficacy of different disinfectants against common healthcare pathogens and found that a combination of bleach and alcohol-based products gave the most effective results. In addition, HPV vapour systems effectively reduce multidrug-resistant organisms in healthcare facilities and significantly reduce environmental contamination from *Clostridioides difficile* spores [16]. UV radiation, particularly UV-C, has gained attention for its ability to damage pathogens’ RNA and DNA, leading to their inactivation [17].

Studies have shown that UV-C technology is effective in eliminating multiple drug-resistant pathogens, including methicillin-resistant *Staphylococcus aureus* (MRSA), vancomycin-resistant Enterococci (VRE) and *C. difficile* [11]. UV-C radiation has also proven to be effective in disinfecting stethoscopes, which are often a source of pathogen transmission [18], and deep environmental disinfection when fitted to mobile robotic equipment [19]. Moreover, UV-C disinfection does not leave any chemical residue, making it a safe and eco-friendly alternative to traditional disinfection methods [20]. Several studies have shown their effectiveness in reducing nosocomial infections in hospitals and healthcare facilities by contributing to disinfecting the air, surfaces and instruments used [21,22].

However, healthcare facilities need to adopt proper procedures to ensure the effectiveness of UV-C disinfection. First, the surfaces to be disinfected must be free of any visible dirt or debris, as these can shield pathogens from UV-C radiation. Therefore, thorough cleaning must precede disinfection. Second, the UV-C device must be positioned appropriately to ensure that all surfaces receive adequate radiation. Third, the exposure time must be sufficient to inactivate the pathogens, which may vary depending on the pathogen type and the UV-C radiation intensity. Finally, proper safety measures must be taken to protect the staff and patients from the harmful effects of UV-C radiation [23].

Due to the limitations of this method of disinfection, research and development of new disinfection technologies are driving new strategies for preventing airborne and cross-contamination infections. A renewed in-depth study of the germicidal properties of the UV spectrum, focusing on the upper and lower limits of this radiation, is attracting increasing interest in the scientific community. For example, violet-blue light in the range of 405–470 nm has shown promising results in disinfecting surfaces and air [24]. This light has been found to have antimicrobial properties that can effectively inactivate a wide range of microorganisms, including bacteria, viruses and fungi [25,26]. The mechanism of action of violet-blue light is based on the production of reactive oxygen species (ROS), which can damage the cellular components of microorganisms and lead to their cellular death [27]. Several studies have demonstrated the effectiveness of violet-blue light in disinfecting surfaces, with a wavelength of 405 nm, reducing bacteria levels on contaminated stainless steel, glass and plastic carriers [28]. One study found that violet-blue light reduced the number of bacteria on surfaces by up to 99.9% after only five minutes of exposure [29]. Another study showed that violet-blue light is effective in reducing the number of microorganisms in the air of a hospital ward, resulting in a decrease in the incidence of HAIs [30].

In addition to its antimicrobial properties, violet-blue light has other advantages as a disinfection method. Unlike some chemical disinfectants, it is safe for humans and does not produce harmful by-products. In fact, the use of light as a physical means of disinfection in hospital environments would minimize the risks of environmental pollution from chemical disinfectants [31].

Moreover, it is cost-effective and easy to use, making it a practical solution for disinfecting surfaces and air in healthcare settings and other environments. Portable devices that emit violet-blue light are available on the market for disinfecting surfaces and air. These devices can be used in hospitals, clinics, laboratories and other settings to reduce the risk of healthcare-associated infections.

The present work aimed to evaluate: (i) the antimicrobial efficacy of violet-blue (405 nm) LED lamps on high environmental contact surfaces in the Hospital Infection Control Laboratory, and (ii) the effect of static irradiation on the bacterial growth of MRSA on various surfaces.

## 2. Materials and Methods

### 2.1. Setting

This pre- and post-experimental study took place in the Hospital Infection Control Laboratory of the Complex Structure of Microbiology of the Hospital of Perugia from September to October 2022. The laboratory in which the experiments were carried out has an area of approximately 14 m^2^, and the lamps used consisted of a square ceiling lamp (60 × 60 cm) equipped with 12 Nichia NVSW219FT white LEDs (Nichia, Anan, Japan) and 69 Luminus SST-10-UV violet-blue light LEDs (Luminus, Sunnyvale, CA, USA) with a wavelength centred at 405 nm and an output of 1.3 watts each. The duration of violet-blue light exposure in the laboratory was 7 consecutive days in a static and closed scenario. Each day, the lamps were switched on from 2.00 p.m., at the end of the work activities, to 8.00 a.m. of the following day. The experiments to assess the microbial reduction of MRSA on the contaminated surface of the laboratory were carried out on an additional day, maintaining the same exposure time as the previous days (18 h). The power consumption per ceiling light for the 18 h of exposure was about 1600 W.

### 2.2. Simulation Model

**Figure 1:**
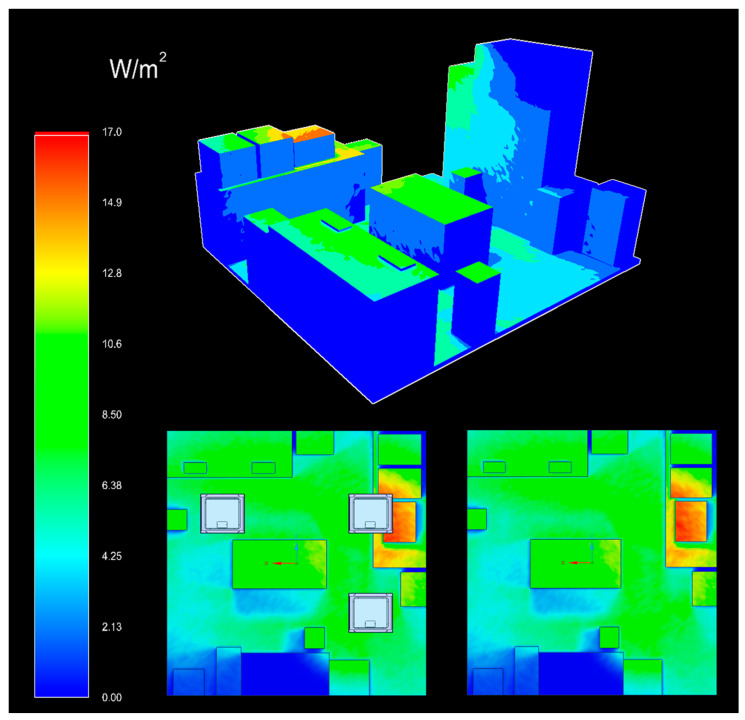
Photoradiometric simulation of the violet-blue light distribution in the laboratory where the tests were carried out. Above is the three-dimensional plane of light distribution, representing intensity with a colorimetric scale associated with irradiance values (in W/m^2^); below left is the section of the room seen from above with ceiling lights and below right without ceiling lights.

The experimental setup for determining the number and location of ceiling lamps in the room where the microbiological tests were carried out was designed using Ansys Speos software (Ansys 2023 R1) (Ansys Inc., Canonsburg, PA, USA). The photometric simulation was realized to ensure good homogeneity of irradiation on all the surfaces in the room equidistant from the ceiling, and to model the spatial distribution of violet-blue light irradiance (Figure 1), always taking into account the structural limitations of the environment in which the tests were carried out. Solidworks 2020 CAD software (Dassault Systèmes, Vélizy-Villacoublay, France) was used to recreate the 3D environment in which the experiment was carried out.

### 2.3. High Touch Surface Sampling

**Figure 2:**
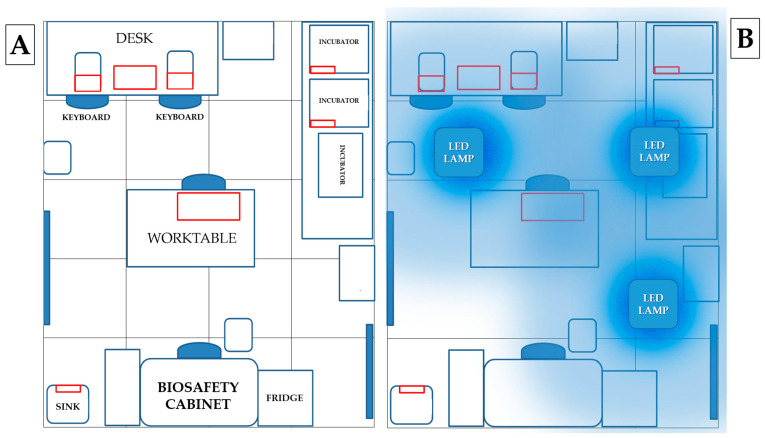
(**A**) Map of sampling points: areas sampled are outlined in red: (i) desk, (ii) keyboard, (iii) incubator handle, (iv) worktable and (v) sink. (**B**) Map of lamps positioned on the ceiling: the three lamps were positioned at the same distance from the perimeter walls of the room in order to illuminate all laboratory surfaces as equally as possible.

High touch surfaces (HTSs) were sampled before (time 0) and after irradiation (time 1–3–5–7 days) with 3 LED lamps positioned on the ceiling of the laboratory. Samples were collected in the laboratory from the desk, worktable, sink, incubator handle and computer keyboard (Figure 2). The heights of the sampled points relative to the floor were, respectively: 90 cm (desk), 75 cm (worktable), 80 cm (sink), 130 cm (incubator handle) and 92 cm (keyboards). Microbiological sampling was performed at 8.00 a.m. before the start of activities (except for day 3, when sampling was performed at 2.00 p.m.) using contact Petri dishes (55 mm Ø), Count-Tact (CT) for bacteria and Count-Tact Sabouraud (CTS) for moulds and yeasts (Biomérieux SA, Marcy-l’Etoile, France) on adjacent non-overlapping surfaces.

The CT and CTS plates contain four neutralising agents to inactivate chemical disinfectants left over from manual cleaning: the combination of lecithin, polysorbate 80 and L-histidine neutralises aldehydes and phenolic compounds; the combination of lecithin and polysorbate 80 neutralises quaternary ammonium compounds, polysorbate 80 neutralises hexachlorophene and mercury derivatives, sodium thiosulphate neutralises halogen compounds and lecithin neutralises chlorhexidine [32]. RODAC plates were pressed on each surface for 10 s and then incubated for 48 h at 37 °C (CT) or five days at room temperature (CTS) [26].

### 2.4. Sampling of Surfaces Contaminated with MRSA

The *S. aureus* used in the study was a clinical MRSA strain, isolated at the Complex Structure of Microbiology of the Santa Maria delle misericordia Hospital in Perugia, on Muller Hinton agar (MHA) plates (Biomérieux SA, Marcy-l’Etoile, France). A single colony was inoculated into Muller Hinton broth (MHB) (Biomérieux SA, Marcy-l’Etoile, France) and maintained at 37 °C for 18 h. The bacterial suspension was then centrifuged and suspended in sterile saline solution to a concentration of 0.5 McFarland. Finally, the inoculum was diluted to a final concentration of 2 × 10^4^/mL.

The bactericidal effect of violet-blue light was evaluated on the non-porous surfaces of (i) plastic (computer keyboard) and (ii) steel (worktable and sink). Aliquots of 100 μL of a bacterial suspension (2 × 10^4^/mL) of MRSA were distributed on selected spots with a surface area of 55 mm diameter. The surfaces were exposed to violet-blue light for 18 h. After irradiation, bacteria were harvested using CT contact Petri dishes (55 mm Ø) (Biomérieux SA, Marcy-l’Etoile, France). As a positive control, parallel areas adjacent to the selected spots were contaminated with the same bacterial suspension and covered with three layers of aluminium to avoid UV exposure. At the end of irradiation, the exposed and covered surfaces were sampled, the plates incubated overnight at 37 °C and the number of colonies counted. All experiments were performed in triplicate.

### 2.5. Statistical Analysis and Data Processing

Microsoft Excel 2016 software was used to organize the empirical data, as CFUs/plate, into a database and for descriptive statistics. Inferential statistical analysis was carried out using Stata 17 software (StataCorp LLC, College Station, TX, USA); the *t*-test was used to assess whether the mean differences in CFUs after irradiation (day 1–3–5–7) compared with CFUs before irradiation day 0, choosing a 95% significance level (*p* < 0.05).

## 3. Results

The results of the photoradiometric simulation showed an irradiance distribution at different points between 1.5 and 10 W/m^2^. We reported the energy dose in W/m^2^ considering that the room’s area was specified in m^2^. In fact, the site with the lowest irradiance (the sink) recorded 1.5 W/m^2^, and the highest (the worktable) was 10 W/m^2^, with an average irradiance of 9 W/m^2^ for the other points (Table 1). Thanks to the data obtained from the simulation, it was possible to calculate the daily energy doses administered over the 7 days and to correlate them with the microbial reduction results at the different points (Table 1).

**Figure 3:**
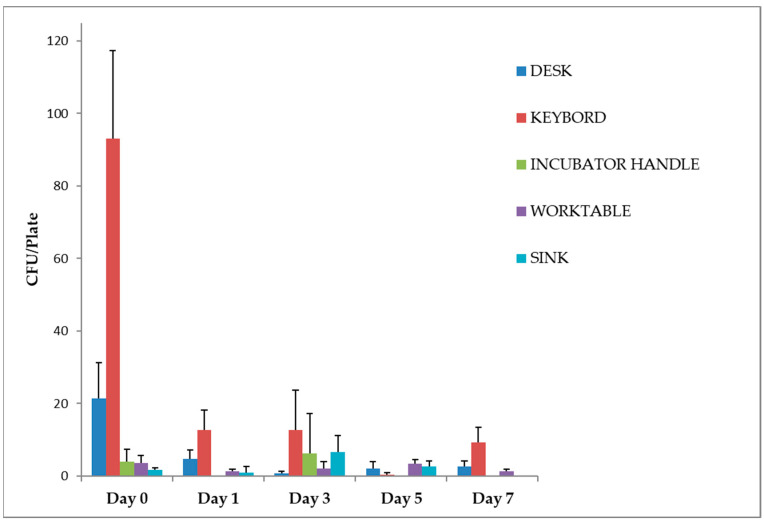
Work surfaces disinfection, irradiated with violet-blue light. Data are expressed as mean ± SD (standard deviation) of three determinations carried out in adjacent areas at the same point. The number of CFU of bacteria, yeasts and moulds was evaluated as described in the Section 2. Statistical analysis of the raw data was performed by *t*-test.

Exposure to violet-blue light reduced the number of CFU of bacteria, yeasts and moulds in all sampled areas after 24 h. In particular, a significant trend (*p* < 0.05) was observed for the desk and the keyboards, with a mean number of colonies on day 1 of 5 (CI 0–10) and 13 (CI 2–23) CFU/plate (reduction to day 0 of 78.13% and 86.38%, respectively), which remained constant until day 7 with 3 (CI 0–6) and 9 (CI 1–17) CFU/plate (87.50% and 89.96%, respectively) (Figure 3).

The incubator handle already showed a low total microbial load (4 ± 3 CFU/plate) at time 0. No microbial growth was detected after irradiation on days 1, 5 and 7. The microbial growth observed on day 3 (6 ± 11 CFU/plate) can be attributed to the time of sampling (Figure 2). This was the only day on which sampling was performed at 2 p.m. after a busy working day during which the incubator was opened several times. Of the three plates used for the control, only one showed colony growth (19 CFU/plate).

For the worktable and sink, the microbial load did not show a significant reduction after irradiation with UV lamps. Still, the low number of colonies found even before disinfection should be considered (Figure 2).

**Figure 4:**
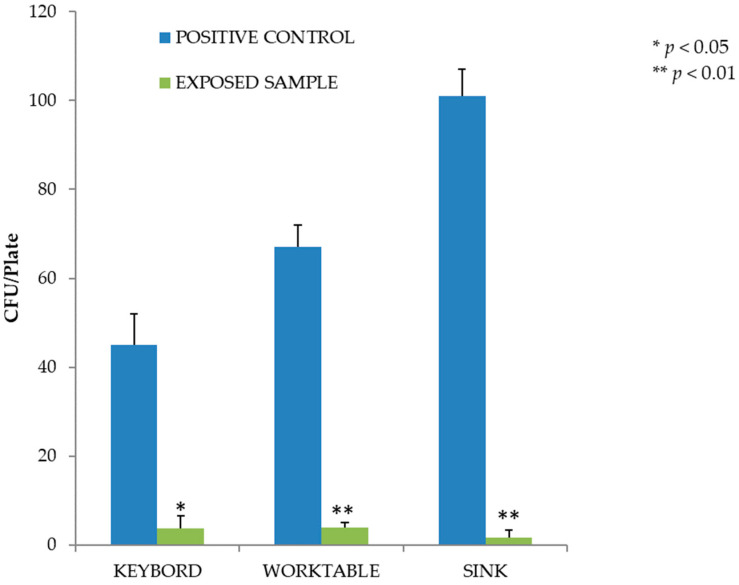
Inactivation of MRSA after exposure to the violet-blue lamp. Data are expressed as mean ± SD (n = 3). * *p* < 0.05 (CFU after irradiation, days 1–3–5–7, vs. CFU before irradiation, day 0).

In the second experiment, we evaluated the effectiveness of LED lamps on MRSA-contaminated surfaces. On all contaminated surfaces, the level of reduction achieved was significant (*p* < 0.05). As shown in Figure 4, for the same concentration of bacterial load on the surface, the highest number of colonies was recovered from steel surfaces (on average 67 CFU/plate, CI 57–77, on the worktable and 101 CFU/plate, CI 89–113, on the sink in the controls), a smoother material than the plastic of the computer keyboard. The percentage reduction in MRSA microbial load on the surfaces compared to the covered controls was 91.8% on the computer keyboard, 94.0% on the worktable and 98.3% on the laboratory sink.

## 4. Discussion

The violet-blue lamps used in our study significantly reduced the total surface microbial load in the environment in which they were installed. The sampled HTS were irradiated for 18 h a day for one week. The different surfaces encountered many different operators, who changed during working hours on the different days of the experiment. Despite this, the data obtained showed a steady reduction in contamination, which remained constant over the days. The only exceptions were the points sampled at the sink and the laboratory workbench, where the contamination level (which was already low to begin with) fluctuated significantly or remained constant compared to time 0. This is probably due to secondary variables, such as objects or materials placed on these surfaces, which formed shaded areas adjacent to the sampled points, preventing the light from completely disinfecting the surface. The correlation between the daily energy doses and the level of reduction achieved at the various points is certainly there, at least as far as the keyboards and the desk are concerned. In fact, looking at the graph in Figure 3, we can see a decreasing trend that remains constant at the points where the daily energy dose was 61.6 and 55.1 J/cm^2^. At the point of maximum and minimum irradiation, where the daily doses were 64.8 and 9.7 J/cm^2^, respectively, it is not possible to establish a direct correlation between the dose and the level of reduction achieved, although it is important to note that the contamination levels remained constantly low during all the days of the experiment. This could be related to the presence of objects shielding the light from the residues of the chemical disinfectants, as mentioned above (surfaces are constantly cleaned during working hours due to the enormous passage of samples and contaminated material), or to the ability of light to inhibit the growth of microorganisms on exposed surfaces. For future experiments, prolonged blue-violet light disinfection of areas such as the keyboard could be investigated. Nevertheless, the results obtained in our study are consistent with those reported in the literature. Indeed, the improvement in the overall cleanliness of surfaces after prolonged exposure to violet-blue light is a finding that has been widely discussed in various studies conducted in different hospital environments. In a study by Murrell et al. [33], the researchers evaluated the effectiveness of violet-blue light in reducing microbial contamination in a hospital orthopaedic operating room. They found that continuous exposure to violet-blue light at a wavelength of 405 nm significantly reduced the total microbial load on surfaces and in the air. The study demonstrated a substantial reduction in bacterial and fungal populations, highlighting the potential of violet-blue light as an adjunctive measure for maintaining a cleaner and safer surgical environment [33]. In a study carried out by Maclean et al. [34] they explored how continuous disinfection with violet-blue light affects the presence of microorganisms, in hospital wards. The researchers incorporated violet-blue disinfection as part of their cleaning protocols. The findings revealed a decrease (by up to 67%) in the microbial count encompassing bacteria and moulds. Similarly, a study by Bache et al. [35] investigated the effectiveness of violet-blue light in reducing microbial contamination in healthcare facilities’ burns unit. The continuous irradiation with light significantly reduced, between 22% and 86%, the mean number of surface bacteria. When the light was no longer being used, there were increases of between 78% and 309%. The study highlighted the potential of violet-blue light to improve the cleanliness and hygiene of office environments, thereby helping to reduce the transmission of healthcare-associated infections [35].

In the second experiment, with MRSA-contaminated surfaces, violet-blue light also significantly affected inactivating the microbial load. The type of surface irradiated was certainly a determining factor. Plastic is known to be a material on which *S. aureus* can easily persist for up to 90 days, with survival time being influenced by factors such as temperature and humidity [36]. Metal is also a non-porous material on which *S. aureus* can easily persist for several weeks. In this case, however, the reflection of light off a reflective surface such as metal may have enhanced the biocidal properties of the radiation. This hypothesis is also supported by the radiation levels at the various contaminated points. For example, although the radiation levels are higher at the keyboard (8.5 W/cm^2^) than at the sink (1.5 W/cm^2^), the metal may have played a key role in promoting the inactivation of surface contamination due to the reflective effect of the material. The higher attenuation values for the worktable than for the keyboards could also follow the same association, but in this case, it is fair to mention that the radiation levels are the same (8.5 W/cm^2^) and that the difference in the results obtained when sampling on the sink could be due to a random factor. The mortality rate recorded after 18 h on the contaminated surface is also worth noting. Indeed, as can be seen from the controls not exposed to light (Figure 3), the average mortality was 1 log_10_ lower than the concentration of the inoculum spread over the laboratory surfaces.

Again, the literature is in agreement with the reduction achieved [37,38,39]. However, the survival rate of *S. aureus* is variable in the literature. There are studies where three to four times less energy is required to achieve the same level of reduction. In a study by Maclean et al. [40], it was evaluated the effectiveness of violet-blue light at different wavelengths, including 405 nm, against MRSA. They found that violet-blue light at 405 nm was highly effective in reducing the viability of MRSA, achieving a reduction of 1 log_10_ with a dose of 15 J/cm^2^. A similar result was obtained in a study by Enwemeka et al. [41] in which, following non-continuous administration of light energy at a dose of 18 J/cm^2^, a reduction of more than 80% of the initial inoculum concentration was achieved. The study also showed that the mode of delivery of the energy dose (continuous or discontinuous) was also important in achieving significant bacterial eradication [41]. This difference is undoubtedly due to the intrinsic differences between different strains of the same species, as has also been reported for experiments with UV-C radiation [42].

Taken together, these studies show that violet-blue light has the potential to be a tool for inactivating microbes. In this sense, our study is a further confirmation of the biocidal capabilities of violet-blue light in a real-world context where the transmission of pathogenic microorganisms through contact with contaminated surfaces and air is a real risk. This frequency can also be used on sensitive healthcare equipment and surfaces [34]. This is not possible with UV-C light, as natural and synthetic polymers undergo significant degradation after prolonged exposure to such radiation [43]. A comparative study of the degradation effects of UV-C radiation and violet-blue light sources on flexible endoscopes showed that germicidal ultraviolet radiation leads to the degradation of the device and an increased risk of infection for patients, unlike violet-blue light [44]. UV-C radiation is absorbed by polymers, resulting in photo-degradation, bond cleavage and chemical transformations that create structural heterogeneity. Cracks formed as a result of photo-degradation have the potential to increase biofouling and inhibit the proper cleaning of instruments and surfaces. In addition, continuous disinfection of healthcare structures in the presence of healthcare workers and patients is not possible with UV-C radiation because chronic exposure to these wavelengths can cause photo-aging, immunosuppression and carcinogenesis in mammalian cells [45]. The set of exposure limit values proposed by the ICNIRP considers hazards in the ultraviolet range from 180 nm to 400 nm, hazards to the retina in the range from 300 nm to 1400 nm of infrared and total radiation hazards to the retina in the range from 380 nm to 3000 nm. They are designated as actinic UV hazards (200 nm to 400 nm), with an effective dose of 30 J/m^2^ for an irradiation period of 8 h. Since optical radiation in the visible and near infrared wavelength range can enter the eye and reach the retina, the hazard is dependent on the size of the luminous surface. This means that large-area light sources with the same illuminance at the eye represent a lower hazard than point-light sources, due to the large area-image on the retina [46]. Mammalian cells and bacteria have different sensitivities to violet-blue light. Studies on osteoblasts suggest that exposure of cells to light at 405 nm up to a dose of 36 J/cm^2^ has no observable effect on cell viability, function, proliferation rate and morphology. Conversely, when the effects of exposure to this wavelength on bacterial cells are studied, results show that the same doses induce significant bactericidal effects [47,48,49].

Despite the almost complete absence of degradative effects on materials and mammalian cells, this type of technology has some important limitations. Although violet-blue light has a higher penetration through transparent materials than UV sources, particularly UV-C, and is also reflected to a greater extent, illumination (and therefore disinfection) of occluded or shaded areas is limited. However, low-intensity doses have the potential to counteract microbial contamination and maintain a healthier environment without the risk of adverse effects on materials or people. Decontamination of spores (e.g., *C. difficile*) requires a much higher dose of light than is permitted in the presence of people. Once again, the ability to exploit the complete absence of physical changes caused by violet-blue light plays a key role in the disinfection process: higher light doses can be achieved by applying lower light intensities for longer periods of time, greatly reducing the photobiological risk to human health. Disinfection can be achieved by combining this frequency with classical decontamination methods. There is a growing conviction that any disinfection method or technology must consider the different boundary conditions and application characteristics, with both advantages and disadvantages. Therefore, the best strategy is the integration of different and complementary techniques, particularly for resistant pathogens (e.g., *C. difficile*) [50]. The introduction of technologies using violet-blue light is precisely in this direction, allowing the development of new processes that are no longer linked to the concept of “occasional and/or periodic” disinfection, but to that of “continuous” disinfection, which is safe, ecological and can also be compatible with the presence of people [51].

Another limitation of violet-blue LEDs is the non-negligible width of the light spectrum around the nominal wavelength: if the peak is at 405 nm, the left tail of the energy falls into the UVA spectrum (wavelength < 400 nm), generating possible photobiological risks. According to CEI EN 62471 [52], it is important to assess the magnitude of this UV-A light component to ensure safe technology. This research addresses the problem both by incorporating technologies that screen out unwanted frequencies and by encouraging chip manufacturers to adapt their products so that they can be easily marketed as compliant with photobiological risk regulations. To date, however, the irradiance of LEDs can be regulated using special sensors that allow the light intensity to be temporarily reduced in the presence of people or pets. In addition, the peak wavelength of LEDs can be increased so that their left tail does not fall on the UV-A spectrum or is so low that it poses no photobiological risk. The introduction of nUV-A disinfection is also a promising scenario, given that (i) the light extraction efficiency of such LEDs is already above 40%, is still increasing and is expected to soon reach that of white light sources (>90%); (ii) the impact of COVID-19 has radically changed our lifestyles, raising awareness of the introduction of hygiene, with an increased need for disinfection systems for air and surfaces (objects and environments); (iii) violet-blue light LEDs have similar energy consumption to standard white LED ceiling lights, allowing them to be used in continuously lit environments, minimizing their economic impact but highlighting their crucial role in disinfection; (iv) the market trend for devices with UV LED sources, which was already growing, has been catalysed by COVID-19 with the introduction of disinfection and cleaning systems from 2019; and (v) the market for these LED devices is already over one billion dollars and projections for 2025 indicate that it will reach three billion dollars [53].

## 5. Conclusions

LED lamps emitting violet-blue light can significantly reduce the environmental microbial load on a range of material surfaces and have been shown to be effective against antimicrobial-resistant bacteria. In fact, in environmental tests, the lamps reduced the number of bacteria, moulds and yeasts on each surface. Light exposure significantly inhibited bacterial growth in vitro. The reduction in the microbial load of MRSA was influenced by the type of material of the non-porous surfaces.

Taken together, this study and the literature references indicate the potential of violet-blue light as an effective strategy for reducing the overall microbial load in various hospital environments, including wards, operating theatres, laboratories and offices. However, it is important to note that violet-blue light disinfection should be considered as an adjunct to standard cleaning practices and infection control protocols.

Further research is needed to optimise the implementation of UV disinfection, considering factors such as duration and intensity of exposure, distance from the light source and compatibility with different surface materials. Nevertheless, the results of these studies provide valuable insights into the potential of violet-blue light as a promising tool for maintaining cleaner and safer hospital environments.

## Figures and Tables

**Table 1:** Photoradiometric simulation results at sampled HTS points.

Position	Height from Floor	Simulated Irradiance	Energy Dose *
	(cm)	(W/m^2^)	(J/cm^2^)
Incubator handle	75	10	64.8
Desk	90	9.5	61.6
Worktable	130	8.5	55.1
Keyboards	92	8.5	55.1
Sink	80	1.5	9.7

* The emitted energy dose is calculated by multiplying the exposure time (18 h) by the irradiance at the sampled point. Therefore, the reported value should be considered as the daily energy dose administered.

## Data Availability

Data are available on reasonable request. The data presented in this study are available on request from the corresponding author.
